# A Study on Job Stress Factors Caused by Gender Ratio Imbalance in a Female-Dominated Workplace: Focusing on Male Airline Flight Attendants

**DOI:** 10.3390/ijerph19159418

**Published:** 2022-08-01

**Authors:** Kieun Lee, Jinyoung Olivia Choi, Sunghyup Sean Hyun

**Affiliations:** School of Tourism, Hanyang University, Wangsimni-ro 222, Seongdong-gu, Seoul 04763, Korea; kieun03@naver.com (K.L.); cjinoung@hanyang.ac.kr (J.O.C.)

**Keywords:** male flight attendants, job stress, mental health, turnover intention, gender differences in communication, relationship conflict with colleagues, hierarchical organizational culture, role overload, gender role conflict, performance appraisal, perceived family support, perceived organizational support, job position

## Abstract

This study investigated the factors that cause job stress among male flight attendants in a female-dominated airline organization, as well as the impact of job stress on their mental health and turnover intention. It also attempted to determine whether perceived family support, perceived organizational support, and job positions had moderating effects on male flight attendants’ job stress. Six job stress factors were identified through focus group interviews and a literature review. A survey was conducted from 1 January to 2 February 2022 to validate the research model, and 188 valid samples were used for statistical analysis. This study discovered that gender differences in communication, relationship conflict with colleagues, hierarchical organizational culture, and role overload had a direct impact on male flight attendants’ job stress. Job stress was found to have a negative impact on mental health and a positive impact on turnover intention. Perceived organizational support was also found to reduce job stress. This study is notably the first to address stress encountered by male flight attendants at work. It offers new directions for future airline personnel management and research. It also presents practical implications, such as the development of training and personnel management programs for male flight attendants.

## 1. Introduction

From 2014 to 2018, nearly 2000 cases of illegal activities on aircraft were reported, including in-flight disturbances, sexual harassment, assault, passenger disputes, and smoking [1]. To address these issues, airlines have proposed ways to improve safety and security, while gradually increasing the proportion of male flight attendants.

Male flight attendant employment is higher in foreign airlines than in South Korean airlines. Air France and Singapore Airlines account for 30% and 40% of male flight attendants, respectively. In contrast, the South Korean domestic airline industry—which includes Korean Air, Asiana Airlines, and national low-cost carriers—currently has 10.55% male flight attendants.

An airline’s cabin crew is a representative product and human resource [2], and the psychological, physiological, and behavioral conditions of service employees are delivered directly to customers, which has a significant impact on customer satisfaction [3]. Male flight attendants face job stress because of a female-dominated work environment and social prejudice, which has a negative impact on their mental health. This is a source of concern when the number of male flight attendants is increasing.

Therefore, this study attempted to identify the effect of job stress on the mental health and turnover intention of male flight attendants, which has not been covered in previous studies, as well as the effects of perceived family support, perceived organizational support, and job position as moderating factors in reducing job stress.

## 2. Literature Review

### 2.1. Male Flight Attendants

Cabin crew, also known as “flowers of the sky”, was originally a male occupation. The majority of the terms and systems used in aviation originated in the marine industry. Since men served food and beverages to passengers on luxury passenger ships, such as the Titanic, service on the airship evolved into a male domain. Daimler Airways, British Airways’ predecessor, hired cabin boys to handle passenger check-ins and baggage at the airport, as well as refreshments and drinks on board. It can be seen that the airport service staff began with men as well. Furthermore, Stout Airlines, the forerunners of Imperial Airlines and United Airlines, and Western Air Express hired male flight attendants in 1926 and 1929, respectively [4].

In South Korea, male flight attendants were deployed under the guise of security personnel until the year 2000. Korean Air did not hire male flight attendants until 2010, but instead filled them through the conversion of existing employees. Currently, Korean Air employs approximately 680 male flight attendants, whereas Asiana Airlines employs 200 male flight attendants. Although this only accounts for approximately 10% of the total number of flight attendants, recruitment is ongoing. The cognitive image of male flight attendants has a positive effect on airline service satisfaction and affinity for customers [5]. Furthermore, the organizational atmosphere has improved for fellow team members who collaborate, resulting in improved job performance.

### 2.2. Job Stress

Research on the effect of job stress on job behavior began in the 1970s, and the terms “organizational stress” and “job stress” were commonly used [6]. Job stress is defined as stress caused by work-related factors [7]. Stress occurs when a situation does not match an individual’s ability or desire to perform a task. Therefore, job stress refers to stress caused at work by work overload, role conflict, and a lack of work autonomy, which endanger employees’ physical and mental health [8].

Research on flight attendant stress has been conducted since the 2000s [2]. As service workers are easily exposed to job stress due to close interpersonal contact and the work environment [9], managing employee job stress is important for effective human resource management. Continuous job stress can lead to problems such as decreased job performance, decreased job satisfaction, increased turnover intention, decreased productivity and innovation, and even depression and suicide [10].

Six job stress factors were identified in this study through a focus group interview with 25 male flight attendants currently employed by K Airlines and a literature review.

#### 2.2.1. Gender Differences in Communication

The difficulty in communication between men and women stems from the fact that they perceive the purpose of conversation differently. Women use communication to strengthen social connections and build relationships, whereas men use language to exert control and achieve tangible results [11,12,13]. Most women use conversation as the language of relationships, whereas most men use dialogue to maintain independence, establish relationships, and negotiate. Male flight attendants experience psychological stress because of communication difficulties in female-dominated work environments.

**Hypothesis** **1** **(H1).** *Gender differences in communication have a positive (+) effect on male flight attendants’ job stress.*

#### 2.2.2. Relationship Conflict with Colleagues

Relationship conflicts among colleagues cause unpleasant feelings such as cynicism, distrust, and hostility among members, focusing on the argument itself rather than the purpose of the argument [14,15]. Flight attendants work as a team, and relationship conflicts are unavoidable in a team’s organizational structure. Relationship conflict with colleagues has been identified as a factor preventing members from being satisfied and immersed in the organization [16,17]. Furthermore, it causes people to spend time and energy focusing on the other person who is the target of the relationship conflict rather than organizational problems, and inhibits the cognitive process between members by increasing stress and anxiety [18,19].

**Hypothesis** **2** **(H2).** *Relationship conflict with colleagues has a positive (+) effect on male flight attendants’ job stress.*

#### 2.2.3. Hierarchical Organizational Culture

A hierarchical organizational culture has a vertical structure with a strong conservative tendency to value subordination, orders, principles, and procedures. This culture has the advantage of improving internal efficiency by strengthening the normative control of organizational members; however, it can reduce job attractiveness by having many formal orders and procedures, and weakening motivation [20,21]. In South Korean airlines, there is a hierarchical culture among cabin crew members that is determined by the year of employment. This is a part of the culture and not a rule imposed by an airline [22].

**Hypothesis** **3** **(H3).** *Hierarchical organizational culture has a positive (+) effect on male flight attendants’ job stress.*

#### 2.2.4. Role Overload

Role overload occurs when too much responsibility and work is assigned for a given amount of time, ability, and context [23]. When members experience role overload, they perceive their job demands to be increasing, and while no changes in job resources are made to address them, they experience job stress and have a negative attitude toward the organization [24]. Role overload in the airline industry refers to work that makes it difficult for flight attendants to keep up with their assigned schedules. Male flight attendants board less than 5% of all flights and, in addition to their work, face overburdening situations such as patient occurrence, disability care, aircraft system failure, and reckless passengers.

**Hypothesis** **4** **(H4).** *Role overload has a positive (+) effect on male flight attendants’ job stress.*

#### 2.2.5. Gender Role Conflict

Gender role conflict is a psychological state in which an individual internalizes gender roles during the socialization process and negatively affects themselves or others [25]. Gender role conflict occurs when one believes that one does not fit the attributes defined in a particular gender role, and the degree of conflict increases when one is criticized by others. Gender role conflict is known to be significantly related to low self-esteem, depression and anxiety, overall psychological loss, and hostility [26], and male gender role conflict has been shown to significantly predict stress levels in previous domestic studies [27,28].

**Hypothesis** **5** **(H5).** *Gender role conflict has a positive (+) effect on male flight attendants’ job stress.*

#### 2.2.6. Performance Appraisal

Performance appraisal has two goals: administrative and developmental. Administrative objectives are directly related to decisions such as pay, promotion, deployment, and dismissal, whereas developmental objectives identify weaknesses and strengths based on feedback from assessment results, and influence employee training and development decisions [29]. In the case of large domestic airlines, the importance of performance appraisal is emerging in many areas, such as promotion and reward management. The performance appraisal system for individual flight attendants includes a flight work evaluation (conducted by each flight secretary), quarterly performance evaluation, and competency evaluation. However, there were numerous complaints from respondents about whether the office manager, as an evaluator, can objectively judge all team members’ work skills in the unique environment of an airplane.

**Hypothesis** **6** **(H6).** *Performance appraisal has a positive (+) effect on male flight attendants’ job stress.*

### 2.3. Perceived Family Support

Family support implies that family members take care of, respect, and value one another through mutual interaction [30]. In particular, psychological and emotional support that gives one the sense that one is receiving enough care and love from one’s family is useful in coping with crises and adapting to change [31]. Several studies have found that family support is a major mediating factor in reducing depression and stress and promoting adaptation [32]. When exposed to stress in a work environment where various stressors are scattered, family support plays a role in overcoming stress in the workplace and living environment, and the higher the family participation, the more effectively the individual can cope with and recover from stress [33].

**Hypothesis** **7a** **(H7a).** *Male flight attendants’ job stress varies depending on perceived family support.*

### 2.4. Perceived Organizational Support

Perceived organizational support refers to an employee’s overall perception that an organization values employee commitment and cares about employee well-being [34]. The importance of organizational support in organizational management has been recognized because an organization’s fair evaluation of employees’ contributions, as well as the resulting welfare support, forms positive or negative attitudes and behavioral responses toward the organization in the future [35]. Perceived organizational support enables employees to achieve performance goals, extra-role behaviors, a sense of accomplishment, and more positive behavior toward their own organization (e.g., increased job satisfaction, positive psychological state, reduced stress and turnover rate) and enables active job performance accordingly [36,37].

**Hypothesis** **7b** **(H7b).** *Male flight attendants’ job stress varies depending on perceived organizational support.*

### 2.5. Job Position

Positions are the most basic level of classification for jobs with similar types of work, difficulty, and responsibility, and different positions exist in the organization. The majority of airline positions begin as general flight attendants and progress to assistant pursers, pursers, and managers (senior and chief pursers). Several studies have found that job position has a significant relationship with various types of stress, job satisfaction, and other related factors experienced by employees while performing their jobs. Wade et al. demonstrated that the ability to control stress increases with job position, resulting in discriminatory turnover intention for each position [38]. This means that the higher the position, the better is the adaptation to the working environment, and the position can be expected to reduce job stress.

**Hypothesis** **7c** **(H7c).** *Male flight attendants’ job stress varies depending on job position.*

### 2.6. Mental Health

Mental health is defined as the ability to form and maintain satisfying human relationships without suffering from mental illness [39]. Employees with mental health issues may have higher absenteeism, lower productivity, poor decision-making, loss of motivation and commitment, conflict with coworkers, and worsening customer relationships [40]. As a result, psychosocial risk assessment should be expanded to protect workers’ mental health, and it should be managed at the organizational level in various ways to conduct job stress risk assessment [41].

**Hypothesis** **8** **(H8).** *Job stress has a negative (−) effect on male flight attendants’ mental health.*

### 2.7. Turnover Intention

Turnover intention refers to an organization member’s intention or attempt to give up being a member of the workplace and leave their current job [42], which includes the act of leaving the organization voluntarily, looking for another job, and contemplating change [43]. The factors influencing turnover intention are classified as overall organizational, working environment, job content, and individual factors. Employee turnover intention, particularly in hospitality industries such as airlines, can have a negative impact on the professionalism and consistency of service quality provided to passengers, as well as on the failure to form long-term relationships with customers [44].

**Hypothesis** **9** **(H9).** *Job stress has a positive (+) effect on male flight attendants’ turnover intention.*

## 3. Research Methods

### 3.1. Research Model and Hypotheses

**Figure 1:**
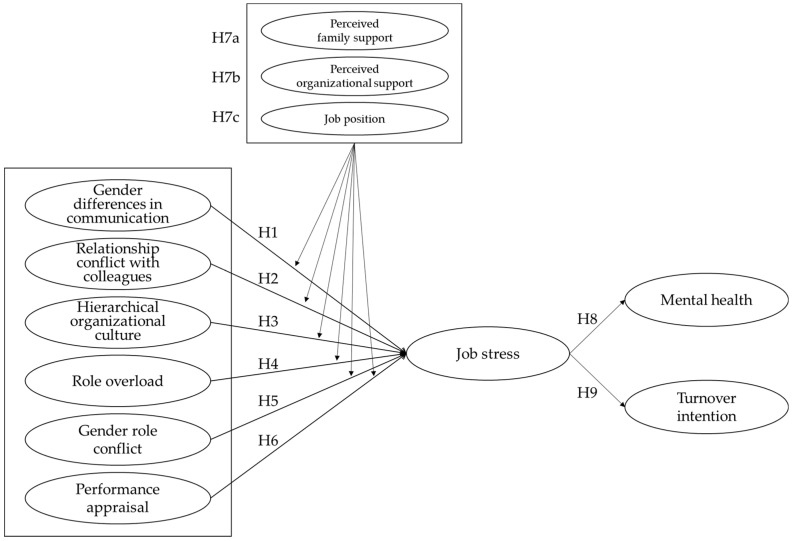
Research model. **Hypothesis 1 (H1).** Gender differences in communication have a positive (+) effect on male flight attendants’ job stress; **Hypothesis 2 (H2).** Relationship conflict with colleagues has a positive (+) effect on male flight attendants’ job stress; **Hypothesis 3 (H3).** Hierarchical organizational culture has a positive (+) effect on male flight attendants’ job stress; **Hypothesis 4 (H4).** Role overload has a positive (+) effect on male flight attendants’ job stress; **Hypothesis 5 (H5).** Gender role conflict has a positive (+) effect on male flight attendants’ job stress; **Hypothesis 6 (H6).** Performance appraisal has a positive (+) effect on male flight attendants’ job stress; **Hypothesis 7a (H7a).** Male flight attendants’ job stress varies depending on perceived family support; **Hypothesis 7b (H7b).** Male flight attendants’ job stress varies depending on perceived organizational support; **Hypothesis 7c (H7c).** Male flight attendants’ job stress varies depending on job position; **Hypothesis 8 (H8).** Job stress has a negative (−) effect on male flight attendants’ mental health; **Hypothesis 9 (H9).** Job stress has a positive (+) effect on male flight attendants’ turnover intention.

This study aimed to investigate the impact of six job stress factors (gender differences in communication, relationship conflict with colleagues, hierarchical organizational culture, role overload, gender role conflict, and performance appraisal) on the mental health and turnover intention of male flight attendants, as well as the moderating effects of perceived family support, perceived organizational support, and job position. Figure 1 depicts the research model developed based on the theoretical and empirical background.

### 3.2. Measurement

Gender differences in communication were assessed using four items from the Down and Hazen Communication Satisfaction Questionnaire (CSQ) [45]. The items on relationship conflict with colleagues comprised four items, using the scale developed by Jehn [46]. Based on Kimberly and Quinn’s theory [47], hierarchical organizational culture consisted of four items. The questionnaire on role overload was reconstructed into four items based on previous studies [48,49]. Gender role conflict was measured using four items based on the Gender Role Conflict Scale developed by O’Neil et al. [50]. Four items were used to assess performance appraisals, based on Lee’s research questionnaire [51].

Based on the research of Quick and Quick [52], the measurement of job stress consisted of four items reconstructed through focus interviews with male flight attendants. The Psycho-Social Well-being Index [53] was used to assess mental health, which consisted of four items. In terms of turnover intention, four items were selected using Mobley’s measurement tool [54]. Four items on perceived family support were created using Kang’s family support measurement tool [55]. The measurement of perceived organizational support consisted of four items, using Yoo’s items [56], which were adapted from the content developed by Eisenberger et al. [57].

All measurement items were assessed using a 5-point Likert scale ranging from 1 (not at all) to 5 (very much).

### 3.3. Data Collection and Analysis Method

The survey was conducted for male flight attendants at domestic K Airlines from 1 January to 2 February 2022, but only for those who had worked for two years or more, excluding interns. A total of 188 valid responses were analyzed, excluding 14 insincere responses.

The methods used to analyze the collected data were as follows. First, a frequency analysis was conducted to understand the demographic characteristics of the participants. Second, a confirmatory factor analysis (CFA) was performed to determine the validity of the latent and observed variables. Third, to evaluate the reliability of the measurement items, the Cronbach’s α coefficient was checked. Fourth, descriptive statistical analysis was performed to identify the levels of the major variables, and skewness and kurtosis were checked to determine whether the data normality assumption was met. Fifth, to verify the causal relationships between the six job stress factors, job stress, mental health, and turnover intention, a structural equation model (SEM) analysis was performed. Finally, a multigroup analysis was conducted to confirm the moderating effects of perceived family support, perceived organizational support, and job position. SPSS 25 and AMOS 25 were used for statistical analysis, and statistical significance was determined using a significance level of 5%.

## 4. Research Results

### 4.1. Demographic Characteristics of Participants

Table 1 presents the demographic characteristics. In terms of participants’ age, those aged 30–39 (69.7%) were the most numerous. Marital status was found to be evenly divided between single (50.0%) and married (50.0%) individuals. The majority of the respondents had a university degree (83.5%). As for length of service, nearly half of the participants had worked for more than 5 years and less than 10 years (48.4%), followed by those with more than 20 years (16.0%). Job positions were AP (39.9%), PS (22.9%), SD (21.3%), CP (10.6%), and SP (5.3%). In terms of annual income, the highest amount was 70 million won or higher (31.4%).

### 4.2. Confirmative Factor Analysis and Reliability Analysis

CFA was performed to determine the validity of latent and observed variables. The results of the analysis are presented in Table 2. The items “I sometimes find it difficult to communicate with female colleagues” (gender differences in communication), “Our organization prioritizes the stability and order over change” (hierarchical organizational culture), “There is no personal break during overseas stay” (role overload), and “I work with tension during flight work” (job stress) were eliminated because the factor loading value was less than 0.50, which was deemed insufficient.

In terms of the measurement model fit, the comparative fit index (CFI) was 0.914, the Tucker–Lewis Index (TLI) was 0.900, and the root mean square error of approximation (RMSEA) was 0.069. CFI and TLI were both greater than the reference value of 0.90, while RMSEA was less than 0.08, indicating that the measurement model was valid. Furthermore, except for the four items mentioned above, all items had a factor loading of 0.50 or higher, and the Cronbach’s α coefficient was greater than 0.70.

### 4.3. Convergent Validity and Discriminant Validity Verification

To validate the convergent validity, which determines the degree of consistency of the observed variable constituting the latent variable, composite reliability (CR) and average variance extracted (AVE) were calculated using factor loading and measurement error. Table 3 presents the results. The CR ranged from 0.825 to 0.951, and the AVE ranged from 0.542 to 0.867. When CR is 0.70 or higher and AVE is 0.50 or higher, it is considered to satisfy the convergent validity condition [58].

Next, the correlation coefficient between latent variables was examined to see if the similarity between variables was excessive, and the results of the correlation analysis are presented in Table 4. The coefficient of correlation between gender role conflict and performance appraisal was the highest at 0.869, and most showed a significant positive (+) correlation, while mental health showed a significant negative (−) correlation with the other variables.

To verify the discriminant validity, the verification method proposed by Bagozzi and Yi [59] was used. As shown in Table 5, the chi-square statistics of the model that merged gender role conflict and performance appraisal with each variable were compared. The degrees of freedom for the two models differed by a factor of 8. If the degrees of freedom differ by 8, a chi-square value of 15.507 or greater indicates a statistically significant difference. The difference in chi-square values between the two models was 77.184, which was much greater than the threshold of freedom 8 (15.507). Consequently, the two models show a statistically significant difference, and it can be concluded that the original model with a relatively low chi-square value is more appropriate. Even when comparing the model fit index, the original model outperformed the merged model.

### 4.4. Descriptive Statistics and Normality Verification

Table 6 shows the results of a descriptive statistical analysis performed to understand the level of the variables. The average gender difference in communication was 2.62, average relationship conflict with colleagues was 2.22, average hierarchical organizational culture was 2.87, average role overload was 2.57, average gender role conflict was 3.40, and average performance appraisal was 3.22. The average gender role conflict was the highest among the independent variables, followed by performance appraisal. Job stress, mental health, and turnover intention averaged at 2.27, 4.03, and 2.27, respectively.

As a result of checking skewness and kurtosis to determine whether the data satisfied the normality assumption, skewness ranged from −0.54 to 0.47, and kurtosis ranged from −0.90 to −0.19. The skewness was less than the absolute value of 2, and the kurtosis was less than the absolute value of 7, indicating that the data met the assumption of normality [60].

### 4.5. Structural Model Analysis and Hypotheses Verification

#### 4.5.1. Model Fit Analysis

Model fitness indices were examined to determine whether the structural model was appropriate. As a result, the CFI was 0.911, 0.900, and 0.069, respectively (see Table 7). CFI and TLI both exceeded the standard value of 0.90, while RMSEA fell below the standard value of 0.08, indicating that the SEM developed for this study is adequate.

#### 4.5.2. Direct Effects Analysis Results

The path coefficients and statistical significance of each path were examined to confirm the structural relationships between variables in the research model (see Table 8). Gender differences in communication (β = 0.260, *p* < 0.05), relationship conflict with colleagues (β = 0.338, *p* < 0.05), hierarchical organizational culture (β = 0.214, *p* < 0.05), and role overload (β = 0.273, *p* < 0.05) were found to have significant positive (+) effects on job stress. Furthermore, job stress had a significant negative (−) effect on mental health (β = −0.769, *p* < 0.001) and a significant positive (+) effect on turnover intention (β = 0.749, *p* < 0.001).

#### 4.5.3. Moderating Effects Analysis

A multigroup analysis was conducted to verify whether there was a statistically significant difference in the influence relationship between variables according to perceived family support, perceived organizational support, and job position. The results of the moderating effects analysis are presented in Table 9.

First, as a result of comparing the model in which all paths were equally constrained and the model without any constraint in the groups with high and low perceived family support, the two models showed a difference of eight degrees of freedom and a difference of 8.290 in the chi-square statistic. At eight degrees of freedom, the threshold of the chi-square value was 15.507, indicating that there was no statistically significant difference. Therefore, it was determined that there was no significant difference in the structural relationships between the variables depending on perceived family support.

The moderating effect of perceived organizational support was confirmed by comparing models in the high and low groups (Table 10). The degrees of freedom of the two models differed by eight, and the chi-square statistic differed by 16.201. When the number of degrees of freedom was eight, the threshold value of the chi-square value was 15.507. Since the difference was greater than the threshold value, there was a statistically significant difference. Specifically, the moderating effect of gender differences in communication on job stress had a significant positive (+) effect on low levels of perceived organizational support (β = 0.329, *p* < 0.05). The moderating effect of relationship conflict with colleagues (β = 0.664, *p* < 0.01) and role overload (β = 0.428, *p* < 0.01) on job stress had a significant positive (+) effect only when the level of perceived organizational support was high.

To verify the moderating effect of job position, it was divided into two groups: below assistant purser (AP) and above purser (PS). Comparing the two models, the degrees of freedom differed by eight, and the chi-square statistic differed by 9.536. There was no statistically significant difference because the chi-square value did not reach the threshold value of 15.507, when the number of degrees of freedom was eight. Therefore, it can be concluded that there is no significant difference in the structural relationships between the variables according to job position.

#### 4.5.4. Summary of Hypotheses Verification

The results of the hypotheses and research model validation are summarized in Table 11. It was found that gender differences in communication, relationship conflict with colleagues, hierarchical organizational culture, and role overload had a significant positive effect on the job stress of male flight attendants. It was also verified that the greater the job stress, the worse the mental health and the greater the turnover intention. Furthermore, perceived organizational support was found to be a moderating variable in the job stress of male flight attendants. To summarize, Hypotheses 1, 2, 3, 4, 7b, 8, and 9 were supported.

## 5. Conclusions and Implications

This study on the job stress of male flight attendants in a female-dominated organization combines qualitative and quantitative research. Through focus group interviews and a literature review, six job stress factors were identified as independent variables: gender differences in communication, relationship conflict with colleagues, hierarchical organizational culture, role overload, gender role conflict, and performance appraisal. This study also aimed to investigate the relationship between job stress and mental health as well as turnover intention. Furthermore, it aimed to determine whether perceived family support, perceived organizational support, and job position had moderating effects on male flight attendants’ job stress.

The theoretical and practical implications of this study are as follows. First, studies on job stress focusing on male flight attendants have not been sufficiently conducted in the tourism industry and no attempts have been made to identify the causal relationship in male flight attendants’ job stress. The fact that this study is the first to address job stress encountered by male flight attendants at work makes it noteworthy. This study provides new directions for future airline personnel management and research.

Second, airline flight attendants are known to be affected by traditional work-related stressors [61]. The findings of this study, on the other hand, indicated that male flight attendants had experienced stressful factors related to relationship and communication difficulties with female colleagues or supervisors. This research can be used to develop a communication training program that will assist flight attendants in understanding workplace conflicts caused by gender differences. A shift in how service occupations are perceived, as well as an increase in the percentage of male flight attendants, is driving interest in male flight attendants. Research on the causes of work-related stress and how to deal with it may aid in career planning and self-exploration.

Third, it was confirmed that male flight attendants’ job stress has a detrimental impact on their mental health and increases their turnover intention. These findings supported previous research that discovered a significant relationship between job stress, mental health, and turnover intention in flight attendants [61,62]. It is critical to set up a system for male flight attendants to actively manage their mental health and find ways to relieve work-related stress through education to reduce their workplace stress factors. For instance, training on “work understanding by gender”, “effective communication”, and “stress management and mental health” should be reinforced, in addition to the current regular safety instruction.

Fourth, K Airlines, which operates as a team system, implemented a team replacement program for employees struggling with workload and coworker problems in their relationships. However, the program is not being carried out as intended because of the unfavorable attitudes of the people in the area, who believe that the team change itself admits that they cannot adjust to their work. To revitalize the program, it is critical that the whole organization actively supports a plan to flexibly operate the schedule measures and team occupancy rate for crew members who are experiencing difficulties at work, through internal publicity and the creation of a free work environment.

Fifth, while hierarchical organizational cultures have the advantage of allowing juniors to absorb practical knowledge from more experienced seniors, they also have the disadvantage of fostering a rigid work environment because of the strict hierarchy between seniors and juniors. Crew positions have a lower promotion rate than other occupations, and because a limited number of team leader positions are available, they are only filled when there is a manpower crisis. It is necessary to implement an open personnel system that ensures promotion opportunities based on ability so that competent crew members can apply for the position of team leader.

Lastly, perceived organizational support was discovered to be a significant factor in reducing job stress among male flight attendants. Those who highly perceived organizational support confirmed that job stress caused by relationship conflict with colleagues or role overload can be moderated. It has been demonstrated that organizational work support, financial support, and psychological stability are major motivators for male flight attendants to persevere in demanding work environments. Male flight attendants are heavily influenced by the organization’s welfare policy, personnel system, and working environment. Based on the findings of this study, it is necessary to actively prepare support and systems for economic and psychological compensation for internal customers at the organizational level.

## 6. Limitation and Future Research

Given the limitations of this study, we suggest the following as a possible direction for future research on flight attendant professionalism. First, as the airline industry’s downturn worsened because of COVID-19, most male flight attendants were surveyed while on leave. Many male flight attendants on extended leaves of absence had higher job aspirations than job stress, so it will be necessary to develop a methodology to control the timing more precisely and strictly by dividing the job stress experienced by respondents into before and after COVID-19.

Furthermore, because the hypotheses were only tested on male flight attendants working for K domestic airlines, it may be challenging to extrapolate and apply the research findings to the broader profession of male flight attendants. The professionalism, career goals, and organizational support of male flight attendants are expected to vary depending on the features and working environment of each airline. To generalize the findings of this study, the sample must be expanded to include male flight attendants working in foreign and low-cost airlines.

## Figures and Tables

**Table 1:** Demographic characteristics of participants.

Variable	Index	Frequency (*n*)	Percent (%)
Age	Under 30	8	4.3
	30–39	131	69.7
	40–49	27	14.4
	Over 50	22	11.7
Marital Status	Unmarried	94	50.0
	Married	94	50.0
Education	University degree	157	83.5
	In graduate school	2	1.1
	Graduate degree	29	15.4
Working Period	More than 2 years and less than 5 years	36	19.1
	More than 5 years and less than 10 years	91	48.4
	More than 10 years and less than 15 years	24	12.8
	More than 15 years and less than 20 years	7	3.7
	Over 20 years	30	16.0
Job Position	SD (Steward)	40	21.3
	AP (Assistant Purser)	75	39.9
	PS (Purser)	43	22.9
	SP (Senior Purser)	10	5.3
	CP (Chief Purser)	20	10.6
Annual Income	Less than KRW 50 million	45	23.9
	KRW 50–60 million	43	22.9
	KRW 60–70 million	41	21.8
	Over KRW 70 million	59	31.4
Total		188	100.0

**Table 2:** Confirmative factor analysis and reliability analysis results.

Factors	Items	M (SD)	Loading	Cronbach’s α
Gender differences in communication (3)	I sometimes find it difficult to communicate with the female team leader.	2.45 (1.07)	0.842	0.891
	I sometimes fail to recognize the sincerity of female employees when communicating.	2.69 (1.12)	0.888	
	I find it difficult to discuss personal issues with female colleagues and supervisors.	2.72 (1.23)	0.839	
Relationship conflict with colleagues (4)	I do not want to work with some members of the team due to personality differences.	2.49 (1.19)	0.694	0.822
	I feel nervous and emotionally unstable in the relationship between team members.	2.18 (1.01)	0.886	
	Our team members have conflicts regardless of their work.	2.06 (0.95)	0.736	
	Our team members do not have high intimacy.	2.15 (0.89)	0.618	
Hierarchical organizational culture (3)	There are numerous unnecessary procedures in flight work.	2.82 (1.09)	0.856	0.872
	The working environment is commanding and vertical.	2.86 (1.13)	0.842	
	It is more important to strictly adhere to service regulations than to be flexible.	2.94 (1.03)	0.802	
Role overload (3)	I always feel pressed for time to complete my tasks.	2.56 (1.13)	0.739	0.828
	There are times when I am asked to do work that is beyond my capabilities.	2.61 (1.04)	0.755	
	I struggle at times because I am asked to work on behalf of a colleague in a high-pressure situation.	2.55 (1.09)	0.870	
Gender role conflict (4)	It is critical for a man to be promoted at work.	3.39 (1.18)	0.833	0.878
	It is necessary for a man to be good at everything.	3.49 (1.14)	0.839	
	I am concerned about how others will perceive my performance at work.	3.55 (1.11)	0.841	
	I avoid expressing my emotions to others because I am a man.	3.19 (1.13)	0.703	
Performance appraisal (4)	I feel a lot of pressure from performance appraisal.	3.12 (1.13)	0.866	0.881
	It is prudent to use sick leave because of performance appraisal.	3.52 (1.28)	0.809	
	I am wary of the manager because of performance appraisal.	3.25 (1.23)	0.840	
	I have been stressed by the irrationality of the performance appraisal system.	2.97 (1.25)	0.735	
Job stress (3)	I am not motivated to work on flights.	2.01 (0.92)	0.798	0.829
	I frequently feel helpless and exhausted from my flight work.	2.60 (1.13)	0.774	
	I am conflicted about what I do at work.	2.19 (1.00)	0.795	
Mental health (4)	I am dissatisfied and depressed.	1.96 (0.90)	0.823	0.883
	I have become agitated and irritable these days.	1.83 (0.85)	0.892	
	I have become disturbed and anxious at night.	1.81 (0.88)	0.858	
	I do not wake up feeling refreshed.	2.28 (1.09)	0.713	
Turnover intention (4)	I would like to work for another company.	2.51 (1.23)	0.887	0.867
	I am considering changing jobs if there is a company with better working conditions.	2.82 (1.35)	0.860	
	I am seriously considering leaving the company.	1.88 (0.92)	0.765	
	If I had a second chance, I would not choose the current company.	1.87 (0.96)	0.663	

χ^2^ = 812.670 (df = 428, *p* < 0.001), CFI = 0.914, TLI = 0.900, RMSEA = 0.069.

**Table 3:** Convergent validity analysis result.

Variables	Composite Reliability (CR)	Average Variance Extracted (AVE)
Gender differences in communication	0.864	0.679
Relationship conflict with colleagues	0.823	0.542
Hierarchical organizational culture	0.855	0.663
Role overload	0.951	0.867
Gender role conflict	0.851	0.590
Performance appraisal	0.838	0.566
Job stress	0.825	0.612
Mental health	0.900	0.694
Turnover intention	0.859	0.607

**Table 4:** Correlation analysis results.

Variables	1	2	3	4	5	6	7	8	9
1. Gender differences in communication	1								
2. Relationship conflict with colleagues	0.825 ***	1							
3. Hierarchical organizational culture	0.723 ***	0.695 ***	1						
4. Role overload	0.743 ***	0.754 ***	0.715 ***	1					
5. Gender role conflict	0.586 ***	0.430 ***	0.495 ***	0.668 ***	1				
6. Performance appraisal	0.673 ***	0.634 ***	0.670 ***	0.722 ***	0.869 ***	1			
7. Job stress	0.837 ***	0.873 ***	0.811 ***	0.818 ***	0.480 ***	0.669 ***	1		
8. Mental health	−0.650 ***	−0.743 ***	−0.528 ***	−0.615 ***	−0.335 ***	−0.510 ***	−0.760 ***	1	
9. Turnover intention	0.670 ***	0.605 ***	0.637 ***	0.611 ***	0.414 ***	0.444 ***	0.756 ***	−0.573 ***	1

*** *p* < 0.001.

**Table 5:** Discriminant analysis results.

Model	χ^2^	df	Δχ^2^	Δdf	CFI	TLI	RMSEA
Original Model	812.670	428	77.184	8	0.940	0.930	0.054
Merged Model	889.854	436			0.898	0.884	0.075

**Table 6:** Descriptive statistics analysis results.

Variables	Range	Average	Standard Deviation	Skewness	Kurtosis
Gender differences in communication	1–5	2.62	1.04	0.12	−0.90
Relationship conflict with colleagues	1–5	2.22	0.82	0.28	−0.46
Hierarchical organizational culture	1–5	2.87	0.97	0.12	−0.52
Role overload	1–5	2.57	0.94	0.14	−0.66
Gender role conflict	1–5	3.40	0.97	−0.54	−0.19
Performance appraisal	1–5	3.22	1.05	−0.26	−0.62
Job stress	1–5	2.27	0.88	0.47	−0.32
Mental health	1–5	4.03	0.81	−0.54	−0.37
Turnover intention	1–5	2.27	0.96	0.33	−0.68

**Table 7:** Structural equation model’s fit analysis results.

χ^2^	df	*p*	CFI	TLI	RMSEA
836.910	441	<0.001	0.911	0.900	0.069

**Table 8:** Direct effects analysis results.

Direct Path	B	SE	β	CR	*p*
Gender differences in communication → Job stress	0.214	0.102	0.260	2.105 *	0.035
Relationship conflict with colleagues → Job stress	0.299	0.132	0.338	2.259 *	0.024
Hierarchical organizational culture → Job stress	0.168	0.074	0.214	2.287 *	0.022
Role overload → Job stress	0.243	0.106	0.273	2.295 *	0.022
Gender role conflict → Job stress	−0.122	0.130	−0.162	−0.942	0.346
Performance appraisal → Job stress	0.051	0.136	0.067	0.373	0.709
Job stress → Mental health	−0.774	0.080	−0.769	−9.652 ***	0.000
Job stress → Turnover intension	1.106	0.112	0.749	9.908 ***	0.000

* *p* < 0.05, *** *p* < 0.001.

**Table 9:** Moderating effects analysis results.

Moderator Variables	Model	χ^2^	df	Δχ^2^	Δdf	CFI	TLI	RMSEA
Perceived family support	Unconstrained Model	1617.524	905			0.852	0.838	0.065
	Constrained Model	1625.814	913	8.277	8	0.852	0.839	0.065
Perceived organizational support	Unconstrained Model	1586.663	905			0.858	0.844	0.064
	Constrained Model	1602.864	913	16.201 *	8	0.856	0.844	0.064
Job position	Unconstrained Model	1513.205	905			0.872	0.860	0.060
	Constrained Model	1522.741	913	9.537	8	0.872	0.861	0.060

* *p* < 0.05.

**Table 10:** Moderating effect of perceived organizational support on job stress.

Path	Low Perceived Organizational Support (*n* = 111)		High Perceived Organizational Support (*n* = 77)	
	β	*p*	β	*p*
Gender differences in communication → Job stress	0.329 *	0.013	−0.203	0.476
Relationship conflict with colleagues → Job stress	0.273	0.231	0.664 **	0.004
Hierarchical organizational culture → Job stress	0.190	0.193	0.358	0.063
Role overload → Job stress	0.206	0.406	0.428 **	0.002
Gender role conflict → Job stress	−0.269	0.431	0.171	0.466
Performance appraisal → Job stress	0.251	0.427	−0.347	0.151

* *p* < 0.05, ** *p* < 0.01.

**Table 11:** Summary of hypotheses verification results.

Hypotheses		Result	Standardized Coefficient (β)
H1	Gender differences in communication → Job stress	Supported	0.260 *
H2	Relationship conflict with colleagues → Job stress	Supported	0.338 *
H3	Hierarchical organizational culture → Job stress	Supported	0.214 *
H4	Role overload → Job stress	Supported	0.273 *
H5	Gender role conflict → Job stress	Not supported	−0.162
H6	Performance appraisal → Job stress	Not supported	0.067
H7a	Moderating effect–Perceived family support	Not supported	
H7b	Moderating effect–Perceived organizational support	Supported	
H7c	Moderating effect–Job position	Not supported	
H8	Job stress → Mental health	Supported	−0.769 ***
H9	Job stress → Turnover intension	Supported	0.749 ***

* *p* < 0.05, *** *p* < 0.001.
